# Multiple Imputation of Missing Race and Ethnicity in CDC COVID-19 Case-Level Surveillance Data

**DOI:** 10.6000/1929-6029.2022.11.01

**Published:** 2022-01-28

**Authors:** Guangyu Zhang, Charles E. Rose, Yujia Zhang, Rui Li, Florence C. Lee, Greta Massetti, Laura E. Adams

**Affiliations:** 1 CDC COVID-19 Response Team, Centers for Disease Control and Prevention, Atlanta, Georgia; 2 Health Resources and Services Administration, Rockville, Maryland, USA

**Keywords:** Multiple Imputation, Missing Data, Race and Ethnicity, Health Equity

## Abstract

The COVID-19 pandemic has resulted in a disproportionate burden on racial and ethnic minority groups, but incompleteness in surveillance data limits understanding of disparities. CDC’s case-based surveillance system contains case-level information on most COVID-19 cases in the United States. Data analyzed in this paper contain COVID-19 cases with case-level information through September 25, 2020, which represent 70.9% of all COVID-19 cases reported to CDC during the period. Case-level surveillance data are used to investigate COVID-19 disparities by race/ethnicity, sex, and age. However, demographic information on race and ethnicity is missing for a substantial percentage of COVID-19 cases (e.g., 35.8% and 47.2% of cases analyzed were missing race and ethnicity information, respectively). Our goal in this study was to impute missing race and ethnicity to derive more accurate incidence and incidence rate ratio (IRR) estimates for different racial and ethnic groups, and evaluate the results from imputation compared to complete case analysis, which involves removing cases with missing race/ethnicity information from the analysis. Two multiple imputation (MI) models were developed. Model 1 imputes race using six binary race variables, and Model 2 imputes race as a composite multinomial variable. Our evaluation found that compared with complete case analysis, MI reduced biases and improved coverage on incidence and IRR estimates for all race/ethnicity groups, except for the Non-Hispanic Multiple/other group. Our research highlights the importance of supplementing complete case analysis with additional methods of analysis to better describe racial and ethnic disparities. When race and ethnicity data are missing, multiple imputation may provide more accurate incidence and IRR estimates to monitor these disparities in tandem with efforts to improve the collection of race and ethnicity information for pandemic surveillance.

## 1. INTRODUCTION

The COVID-19 pandemic has disproportionately affected several racial and ethnic groups, with disparities reported in the number of cases, hospitalizations, and deaths [1–4]. However, race and ethnicity are infrequently reported, creating challenges in data analysis and interpretation [5]. Missing data on race and ethnicity are a common challenge in health and health care data systems, despite efforts to improve the accuracy of data collected [6–8]. Missing data for race and ethnicity is a key barrier in monitoring and addressing health disparities among racial and ethnic groups. The CDC case-based surveillance system includes data reported by state and local health departments including case-level information on most COVID-19 cases in the United States (https://www.cdc.gov/coronavirus/2019-ncov/downloads/pui-form.pdf). Information on race and ethnicity is missing for a large proportion of COVID-19 cases reported to the CDC, with 35% missing as of January 13, 2022 (https://covid.cdc.gov/covid-data-tracker/#demographics, last accessed January 14, 2022). Incomplete data on race and ethnicity limits thorough and accurate investigation of racial and ethnic disparities in COVID-19 incidence.

Though a common practice, complete case analysis (removing subjects with missing race/ethnicity information from calculations) may yield biased results [9]. Recent studies have explored alternative methods to complete case analysis to address missingness in race and ethnicity. For example, Fiscella *et al*., [10] and Elliott *et al*., [11–12] developed Bayesian Surname and Geocoding and Bayesian Improved Surname and Geocoding (BISG) methods to estimate the posterior probability of an individual belonging to a given racial/ethnic group using U.S. Census geospatial and U.S. Census surname data. Grundmeier *et al*., [13] developed a Multiple Imputation (MI) model which included the posterior probability of racial/ethnic membership derived from the BISG method as well as demographic and clinical characteristics related to an individual’s race/ethnicity. Ma *et al*., [14] explored MI methods to impute missing race and ethnicity with ZIP code-level information (e.g., racial distribution and income) and individual-level information (e.g., age and mortality) as covariates. Kim *et al*., [15] imputed missing race and ethnicity information using deep learning methods with around 15,000 features including demographic information and clinical events. Labgold *et al*., [16] applied quantitative bias analysis (i.e., BISG based imputation followed by probabilistic bias analysis) to account for missing race and ethnicity.

Using the MI method to impute missing race and ethnicity information has several advantages. First, it can include variables related to race and ethnicity, as well as variables related to missingness on race and ethnicity. Second, MI creates several imputed data sets; the variability within and between the imputed datasets reflects the uncertainty about the missing data. Third, MI techniques have been widely used for several decades and can be performed using standard statistical software, e.g., SAS, R, MICE, STATA. Sequential regression multivariate imputation (SRMI) is a commonly used strategy to construct an imputation model [9, 17–23]. It constructs an imputation model for each variable with missing data. It is flexible and can include variables of different distributions. Imputation methods described in this paper follow the SRMI approach.

Our purpose was to develop MI models to impute missing race and ethnicity information in the CDC COVID-19 case-level surveillance data and evaluate the performance of these models regarding the incidence and incidence rate ratio estimates of COVID-19 cases by race/ethnicity. Two MI models were constructed—one where race is imputed using six binary variables and one where race is imputed as a composite multinomial variable—and applied to the case-level surveillance data. Then, an evaluation study assessed the performance of these models.

## 2. MULTIPLE IMPUTATION OF MISSING RACE AND ETHNICITY IN CDC COVID-19 CASE-LEVEL SURVEILLANCE DATA

### 2.1. Missing Data on Race and Ethnicity in CDC COVID-19 Case-Level Surveillance Data

Our analysis used case-level surveillance data reported from January 20, 2020, through September 25, 2020. Race was reported as one or more of the following race groups: Black, White, Asian, American Indian/Alaska Native (AIAN), Native Hawaiian/Other Pacific Islander (NHPI), and Other race. Records with two or more race groups selected were categorized as multiple race; records with no race categories selected were categorized as missing race. Ethnicity was reported as either Hispanic/Latino or Non-Hispanic/Latino; records with no ethnicity categories selected were categorized as missing ethnicity. Table S1 in the supplemental materials describes the number of COVID-19 cases reported and the percentage of cases with missing race and ethnicity information for each of the 50 states and the District of Columbia (DC). Missingness of race and ethnicity varied by state, with missingness of race ranging from 0.49% (DC) to 99.99% (OK), and missingness of ethnicity ranging from 10.11% (WV) to 99.99% (OK). Overall, missingness of race was 35.82%, and missingness of ethnicity was 47.24%. Race and ethnicity were imputed as two separate variables, i.e., not combined into one variable because missing data patterns differed by race and ethnicity.

### 2.2. Multiple Imputation Models

Our imputation models assumed race and ethnicity data were missing at random (MAR) (i.e., the probability of missingness was not related to the missing data but was related to some of the observed data). To increase the plausibility of the MAR assumption, Little and Rubin [9] recommend including numerous covariates related to the missingness and/or the response variable(s) in the imputation model. Variables related to race and ethnicity as well as variables related to the missingness of race and ethnicity information were identified and included in the imputation model. Case-level surveillance data contains demographic, exposure, and clinical information on each case including age, sex, medical conditions and risk behaviors, clinical course, and symptoms, which could be closely associated with the probability of being a COVID-19 case by different race or ethnicity groups. However, most of this information is missing for more than 80% of the cases, which limits their usability in the MI model. Thus, we included only the case’s age (0.2% missing) and sex (2.0% missing) variables in the MI. Case-level surveillance data also contain information on an individual’s county of residence, state of residence, and Federal Information Processing Standards (FIPS) information. It would be desirable to include data at a finer resolution, e.g., zip code, in the prediction of missing race/ethnicity. However, using a finer resolution such as census block or zip code was not feasible given the lack of finer resolution information in the case-level surveillance data. The five-digit FIPS code can be used to link the case-level data to county-level datasets to predict the probability of individual-level characteristics of the residents in the geographic areas. To improve the MI model, three county-level datasets were linked to the case-level surveillance data using FIPS code: County Health Rankings (2018), Vintage county population data (2018), and CDC/ATSDR Social Vulnerability Index (SVI) data (2018), the latest data available at the time of our study. These county-level datasets included demographics, census information, and CDC SVI. We conducted exploratory data analysis using linear and logistic regression models to evaluate the associations between these county-level variables and race and ethnicity, as well as the missingness of race and ethnicity. We selected 18 county-level variables for inclusion in the imputation model. Table 1 contains the means and percentages of variables selected for the imputation model by race. Tables S2 and S2-A in the supplemental materials contain means and percentages of these variables by ethnicity, and by missingness on race and ethnicity.

Two MI models to impute race were constructed. Model 1 used six binary (Yes/No) race variables and imputed each race variable (Black, White, Asian, American Indian/Alaska Native, Native Hawaiian/Other Pacific Islander, Other) separately. After imputation, an individual’s race was categorized based on the imputed values of these six variables. If only one of the six variables was imputed as “Yes” then the individual’s race was defined by the variable with “Yes”. If more than two race variables were imputed as “Yes” then the person was defined as Multiple race. Multiple race and other race were combined into Multiple/other race category for analysis. Model 2 treated race as a multinomial variable with six categories (Black, White, Asian, American Indian/Alaska Native, Native Hawaiian/Other Pacific Islander, Multiple/other) and imputed the missing values as one of the six categories directly from the model.

For both models, ethnicity was imputed as a binary variable with two levels (Hispanic/Latino and Non-Hispanic/Latino). The MI models were implemented using SAS Proc MI with the fully conditional specification procedure [24]. The discriminate function was used for categorical variables and a regression model for continuous variables. Ten imputations were conducted for each imputation model. After MI, race, and ethnicity were combined into a single analysis variable as Hispanic/Latino, Non-Hispanic White (NH White), Non-Hispanic Black (NH Black), Non-Hispanic Asian (NH Asian), Non-Hispanic American Indian/Alaska Native (NH AIAN), Non-Hispanic Native Hawaiian/Other Pacific Islander (NH NHPI), and Non-Hispanic Multiple/other (NH Multiple/other).

Statistical analysis was conducted separately for each imputed dataset. Incidence (per 1,000) and incidence rate ratio (IRR; NH White as the reference group) were estimated by race/ethnicity over the 50 states and DC for each imputed dataset. State to state variability was accounted for by treating the state and DC as a cluster in a Poisson model (using GEE to account for the clustering) and implemented using SAS Proc GENMOD procedure with the population size as the offset. Results were combined using Rubin’s MI combining rules for the 10 imputation datasets using the SAS MIANALYZE procedure [25–29]. These results were compared with those from the complete case analysis.

### 2.3. Results from Multiple Imputation Models and Complete Case Analysis

Incidence per 1,000 population and incidence rate ratios by race/ethnicity were assessed from the complete case analysis and two MI models (Table 2). Case-level surveillance data contained 49.39% missingness on race/ethnicity (35.82% and 47.24% missingness on race and ethnicity, respectively); as a result, the incidence estimates based on complete case analysis yielded incidence estimates by race/ethnicity approximately 50% lower than those based on the MI data. Based on the complete case analysis, all race/ethnicity groups except NH Asian had a higher risk of COVID-19 compared to NH White, with IRR estimates ranging from 2.13 (95% confidence interval (CI) = 1.88, 2.42) (NH Black) to 3.06 (95% CI = 2.30, 4.07) (NH Multiple/other). The two imputation models yielded incidence and IRR estimates similar to each other, where all groups except NH Asian had higher IRR compared to NH White, with IRR ranging from 1.84 (95% CI = 1.53, 2.22) among NH Black to 4.47 (95% CI = 2.26, 8.87) among NH Multiple/other based on Model 1, and from 1.94 (95% CI = 1.61, 2.33) among NH Black to 5.13 (95% CI = 2.35, 11.22) among NH Multiple/other based on Model 2. The IRR estimates based on the imputation models were higher than the complete case IRR results in two groups, NH Multiple/other and NH NHPI; however, the statistical significance levels of complete case analysis remain unchanged after MI (e.g., among NH NHPI, IRR = 2.99, 95% CI = 1.65, 5.41 from complete case analysis; IRR = 4.18, 95% CI = 2.28, 7.66 from Model 1; IRR = 3.95, 95% CI = 2.11, 7.39 from Model 2).

## 3. EVALUATION OF MI MODELS

An evaluation was conducted to assess the performance of the two MI models. Case-level surveillance data from Minnesota (MN) and Utah (UT) were used because the percent missing race and ethnicity were low (MN: race (11.9%) and ethnicity (16.7%); UT: race (9.8%) and ethnicity (10.9%)); and the case-level data from MN and UT had different race and ethnicity compositions (e.g., MN case data had lower percentages of individuals in the American Indian/Alaska Native (0.83%), Native Hawaiian/Other Pacific Islander (0.22%) and Hispanic/Latino (24.5%) groups, while UT case data had more than 2% of individuals in each race category and 46.1% of individuals in the Hispanic/Latino group). A total of 138,965 cases reported from MN and UT were assessed; 114,793 (83%) had complete age, sex, race, and ethnicity and were used to estimate the “true” incidence and IRR for comparison when conducting the MI and complete case analyses.

Using the target population (MN/UT, N=114,793), two levels of missingness for race and ethnicity (low (i.e., less than 20% missingness) and high (i.e., more than 40% missingness)) were generated as evaluation datasets, assuming MAR missingness. For the low percent of missingness (Evaluation 1), separate logistic regression models were first fitted for missing race and ethnicity using data from Iowa (IA). IA was selected to derive parameters of MAR propensity models since it has low percentages of missingness on race (14.6%) and ethnicity (13.9%). The response variable was whether a subject had missing race and missing ethnicity. Age, sex, and 18 county-level variables (Table 1) were included as predictors, but only variables with p-values ≤0.05 were retained in the final models. These parameter estimates were then used to calculate the probabilities of missing race and missing ethnicity for each person in the MN/UT population. Probabilities were compared with two randomly generated numbers from a Uniform (0, 1) distribution to decide if an individual has missing race and/or ethnicity. More details of the evaluation study can be found in Part 1 of the supplemental materials. Applying IA propensity models to the MN/UT population, 15% of subjects had missing data on race, 17.3% subjects had missing data on ethnicity, 26% subjects had missing values on combined race/ethnicity, on average. This procedure was then repeated using data from Pennsylvania (PA) to fit the propensity models (high percent of missingness; denoted as Evaluation 2). Applying propensity models from PA to the MN/UT population, approximately 44.1% of subjects had a missing race, 53.6% subjects had missing ethnicity, and 64.1% subjects had missing race and ethnicity.

This evaluation used the two aforementioned MI models to impute missing values on race and ethnicity with data from MN/UT. For each evaluation study, the procedure was repeated 100 times (i.e., each time two random numbers were generated for an individual to determine if the individual would have missing values on race and/or ethnicity) to create 100 replicates, and for each replicate 10 imputations were conducted. Incidence per 1,000 by race/ethnicity and IRR by race/ethnicity (with NH White as the reference group) were calculated using a Poisson model with GEE as the method to account for clustering within a county and the log of the county population as the offset. The parameter estimates for the MN/UT target population (i.e., before generating missing values) served as the reference standard. For comparison, incidence and IRR were estimated using the complete case analysis (MN/UT data after generating and removing missing values from the analysis). For MI data, the Poisson model was fit to each imputed data, and the final estimates using the 10 multiply imputed datasets were derived using Rubin’s combination rule. Bias (the difference between the target “true” value derived from the target population and the estimated value using MI or the complete case analyses), relative bias (100*bias/true value), mean width of 95% CI (i.e., the difference of the upper bound and the lower bound of 95% CI) over 100 replicates and coverage rate were then calculated. Coverage was defined as one if the 95% CI covered the true value and zero otherwise.

Results of Evaluation 1 are shown in Table 3. The incidence estimates ranged from 7.17 (NH White) to 64.90 (NH NHPI) per 1,000 for the target population. The complete case analysis yielded results with large biases across all race/ethnicity groups. Biases ranged from −21.55 (NH NHPI) to −2.08 (NH White) per 1,000; relative biases ranged from −52.70% (NH AIAN) to 22.49% (Hispanic/Latino). Coverage rates were zero for all race/ethnicity groups. Using individual race variables (Model 1), MI yielded incidence estimates close to the target population, with biases ranging from −0.60 (NH NHPI) to 1.32 (NH Multiple/other) per 1,000, and relative biases ranging from −3.96% (NH AIAN) to 10.5% (NH Multiple/other). Coverage rates were one for all race/ethnicity groups, which meant the 95% CIs of all the 100 replicates covered the targeted estimates. Using the combined race variable (Model 2), MI yielded incidence estimates with slightly larger biases versus Model 1, with biases ranging from −2.51 (NH Multiple/other) to 2.00 (NH NHPI) per 1,000, and relative bias ranging from −19.97% (NH Multiple/other) to 7.54% (NH Asian). Coverage rates were one for all race/ethnicity groups except the NH Multiple/other group, which had a coverage of 0.18.

The true IRRs ranged from 1.72 (NH Asian) to 9.05 (NH NHPI) for the target population. Though there were large biases in terms of the incidence estimates, the IRR estimates based on complete case analysis were closer to the target population, with biases ranging from −0.61 (NH AIAN) to 0.44 (Hispanic/Latino), and relative biases ranging from −33.33% (NH AIAN) to 9.09% (Hispanic/Latino), and only one group, NH AIAN, with a coverage rate of zero. The IRR estimates for the MI data in Model 1 were close to the target population with biases ranging from −0.08 (NH NHPI) to 0.18 (NH Multiple/other) and relative biases ranging from −3.83% (NH AIAN) to 10.29% (NH Multiple/other). Coverage rates equaled to one for all race/ethnicity groups for Model 1. The IRR estimates using imputation Model 2 were similar to Model 1, with biases ranging from −0.37 (NH Multiple/other) to 0.18 (NH NHPI) and relative biases ranging from −21.14% (NH Multiple/other) to 6.40% (NH Asian). For Model 2, coverage rates equaled to one for all race/ethnicity groups except NH Multiple/other group, which had a coverage of zero.

Table 4 shows the results of Evaluation 2, where the missing percentages on race and ethnicity were higher than those of Evaluation 1. The complete case analysis yielded larger biases in incidence estimates compared to Evaluation 1 because more subjects were removed from the analysis, with biases ranging from 27.53 (NH NHPI) to −4.85 (NH AIAN) per 1,000, relative biases ranging from −85.52% (NH Black) to −36.94% (NH AIAN), and two groups (Hispanic/Latino and NH AIAN) with coverage rates equal to one and remaining groups with coverage rates equal to zero. Multiple imputation Model 1 reduced biases for all groups except the NH Multiple/other group. Excluding NH Multiple/other group, biases using Model 1 ranged from −3.41 (NH NHPI) to 3.80 (NH Asian) per 1,000, relative biases ranged from −22.09% (NH AIAN) to 30.82% (NH Asian), and coverage rates were ≥ 0.95. The NH Multiple/other group had a bias of 9.58 per 1,000. Multiple imputation Model 2 imputed the combined race variable, the bias for NH Multiple/other group (−3.28 per 1,000) was smaller compared to that of imputation Model 1. However, it yielded a lower coverage for the NH Multiple/other race group due to the narrow CI. The narrow width of CI suggests the incidence estimates across county are similar based on imputation Model 2, which leads to a small variance and a lower coverage rate. For the remaining groups, the results of Model 2 were close to Model 1.

The complete case analysis of Evaluation 2 yielded larger biases on IRR estimates compared to Evaluation 1, with biases ranging from −2.27 (NH Black) to 7.58 (NH NHPI) and relative biases ranging from −53.79% (NH Black) to 101.64% (NH AIAN). The MI Models 1 and 2 reduced biases and improved coverage for all groups except NH Multiple/other group.

Evaluations 1 and 2 assume data are missing at random. However, not all variables related to the missingness may be included in the propensity models due to limited individual-level information available in the case-level data. Moreover, it is possible that the missingness of race and ethnicity still depends on race and ethnicity after controlling all possible covariates, i.e., not missing at random (NMAR) missingness. To address this issue, a sensitivity analysis was conducted, repeating the Evaluation studies 1 and 2, with indicator variables on race and on ethnicity included in the propensity models to generate not missing at random missing data. Results found that Model 1 consistently reduced biases of complete case analysis and improved coverage for six race/ethnicity groups, except for the NH Multiple/other group. Imputation Model 2 yielded estimates with slightly larger biases compared to those of imputation Model 1. More details of the sensitivity study can be found in Part 2 of the supplemental materials.

## 4. DISCUSSION

COVID-19 case surveillance provides valuable information on pandemic monitoring to inform public health efforts for epidemic control. Demographic information on race and ethnicity in case surveillance is essential to accurately assess racial and ethnic disparities in COVID-19 incidence and direct efforts to promote health equity. However, high levels of missing data on race and ethnicity and variation in missingness by state constrain the interpretability of estimates of disparities. Removing subjects with missing race/ethnicity information from the analysis may yield biased results and reduce statistical power for detecting health disparities.

To improve estimates and monitoring of COVID-19-related measurements, different methods for grouping persons by race and ethnicity have been explored by Yoon *et al*., [5]. Their analysis shows that different grouping methods can lead to different conclusions about disparities when analyzing race and ethnicity data with missing values. Additional methods for analyzing incomplete race and ethnicity data are necessary to more accurately estimate race and ethnicity incidence and differences. Our study used MI to address the missing race/ethnicity in the case-level surveillance data. We merged county-level information on race/ethnicity distribution, population, and social and economic conditions to the COVID-19 case-level data to construct two MI models. A detailed evaluation of these models found incidence and IRR estimates from Model 1 (imputing race using 6 individual variables) were closer to the true estimates for six race/ethnicity groups (all except the NH Multiple/other). Additional research is needed to identify methods of MI to better estimate race/ethnicity for NH Multiple/other, which was over and under imputed in our two models. In our current analysis race Other was grouped with race Multiple as Multiple/other. An alternative, to be investigated, is to code race Other as the following. First, if race Other is the only race coded as “Yes” then treat it as missing. Second, if race Other and one additional race are coded as “Yes” then categorize the person’s race as the additional race. Third, if race Other is classified as “Yes” and two additional races are classified as “Yes” then categorize the person’s race as Multiple. In future research, the impact of this revised race algorithm should be explored. Our research highlights the importance of collecting complete race/ethnicity information for pandemic surveillance. When missing data exist, MI provides better incidence and IRR estimates to monitor health disparities among racial and ethnic groups. Our MI approach could be adapted to other surveillance data with similar statistical needs, such as COVID-19 vaccination data and other pandemic case-level data.

Our study has some limitations. First, MI model 1 tends to over impute NH Multiple/other group, which includes records with multiple race categories selected or Other race selected. Among the Hispanic/Latino group, the percentage of individuals with Other race selected was much higher compared to the Non-Hispanic/Latino group (32% vs. 4.3%, respectively). Although we included ethnicity as a covariate when imputing race, the higher percentage of Other race among the Hispanic/Latino group might lead to over imputation of Other race among the Non-Hispanic/Latino group. Imputing multiracial identities has previously been reported as a challenge [12] and deserves further investigation. Second, in the case-level data, most of the person-level information is missing and can’t be included in the imputation model. County-level information from census data was merged to perform imputation, which was demonstrated to be effective but may not be the best predictors of individual race and ethnicity. However, it has been shown to perform well in predicting the distributions of race and ethnicity at the aggregated group level [14]. Third, because the completeness of COVID-19 reporting and race/ethnicity missing rates vary by state, the assumption of MAR may not hold for some states. In Evaluations 1 and 2, we used propensity models from IA and PA to generate low and high levels of missing values, but the true missing data mechanism across states may be more complicated than IA and PA propensity models.

In summary, for national case reporting data with a large number of missing values for race/ethnicity, using MI can dramatically reduce the biases in incidence and IRR estimates compared to complete case analysis. Imputing source variables for race separately was more accurate than imputing race directly as a composite variable, which was also a recommended imputation strategy for composite categorical variables in a recent study [30]. Our research highlights the significant problems with incomplete race/ethnicity information for pandemic surveillance. Multiple Imputation resulted in more accurate incidence and incidence ratio estimates for different race/ethnicity groups. It can help fill critical gaps in cases surveillance completeness for race and ethnicity and should be considered to provide more accurate estimates for incidence and IRR in the COVID-19 pandemic.

## Supplementary Material

supplemental material

## Figures and Tables

**Table 1:** Means and Frequencies of Variables in the Multiple Imputation Model by Race

Variables in the imputation model	American Indian/Alaska Native (N=39,626)		Asian (N=103,584)		Black (N=578,104)		Multiple/other (N=628,396)		Native Hawaiian/Other Pacific Islander (N=14,730)		White (N =1,692,333)	
	Mean	SE	Mean	SE	Mean	SE	Mean	SE	Mean	SE	Mean	SE
Food environment index (raw value)	6.41	0.01	8.15	0.00	7.37	0.00	8.08	0.00	7.91	0.01	7.87	000
Limited access to healthy foods (raw value)	0.14	0.00	0.04	0.00	0.06	0.00	0.04	0.00	0.05	0.00	0.06	0.00
Log of median household income (raw value)	10.80	0.00	11.09	0.00	10.90	0.00	11.03	0.00	11.01	0.00	10.96	0.00
Percent Not Hispanic, White alone	48.16	0.11	50.04	0.06	52.20	0.02	49.04	0.02	60.14	0.16	62.30	0.02
Percent Not Hispanic, Black or African American	6.38	0.05	12.21	0.03	25.65	0.02	12.20	0.01	7.65	0.07	11.56	0.01
Percent Not Hispanic, American Indian/Alaska Native	20.68	0.13	0.40	0.00	0.42	0.00	0.51	0.00	0.70	0.01	0.59	000
Percent Not Hispanic, Asian alone	3.03	0.02	11.72	0.03	5.01	0.01	8.86	0.01	7.26	0.06	4.91	0.00
Percent Not Hispanic, Multiple/other race	2.20	0.01	2.74	0.01	1.92	0.00	2.23	0.00	2.59	0.01	1.98	0.00
Percent Hispanic/Latino	19.38	0.07	22.48	0.05	14.73	0.02	26.95	0.02	20.90	0.11	18.51	0.01
Percentile ranking for SVI Socioeconomic	0.63	0.00	0.39	0.00	0.51	0.00	0.47	0.00	0.39	0.00	0.43	0.00
Percentile ranking for SVI Household Composition	0.54	0.00	0.21	0.00	0.37	0.00	0.27	0.00	0.24	0.00	0.33	0.00
Percentile ranking for SVI Minority Status/Language	0.84	0.00	0.89	0.00	0.82	0.00	0.89	0.00	0.83	0.00	0.76	0.00
Percentile ranking for SVI Housing / Transportation	0.76	0.00	0.71	0.00	0.70	0.00	0.72	0.00	0.68	0.00	0.62	0.00
Log of population density	4.29	0.01	7.30	0.00	6.81	0.00	6.89	0.00	6.46	0.01	6.17	0.00
Adult obesity (raw value)	0.30	0.00	0.26	0.00	0.29	0.00	0.26	0.00	0.27	0.00	0.28	0.00
Children in poverty (raw value)	0.26	0.00	0.18	0.00	0.23	0.00	0.20	0.00	0.17	0.00	0.19	0.00
Children in single parent household	0.39	0.00	0.32	0.00	0.40	0.00	0.34	0.00	0.30	0.00	0.33	000
Food insecurity (raw value)	0.17	0.00	0.13	0.00	0.16	0.00	0.13	0.00	0.13	0.00	0.13	0.00
Age	38.95	0.10	44.46	0.06	44.19	0.03	39.08	0.02	37.47	0.15	44.60	0.02
Sex (% male)	46.20	0.25	48.07	0.16	44.51	0.07	49.04	0.06	47.95	0.41	47.67	0.04

Note: P-values <0.001 for all variables for testing if means are equal across six race groups.

**Table 2:** Incidence per 1,000 and Incidence Rate Ratio (IRR) Estimates Based on Complete Case Analysis and Multiple Imputation from Case-Level Data Across 50 States and DC

	Complete case analysis		Multiple imputation model 1 (6 individual race variables)		Multiple imputation model 2 (1 multinomial race variable)	
Race/ethnicity	Incidence (95% CI)	IRR (95% CI)	Incidence (95% CI)	IRR (95% CI)	Incidence (95% CI)	IRR (95% CI)
Hispanic/Latino	12.10 (6.31, 23.20)	2.34 (1.32, 4.13)	23.30 (14.56, 37.29)	2.06 (1.25, 3.41)	23.30 (14.45, 37.57)	2.13 (1.28, 3.52)
NH White	5.18 (4.26, 6.30)	Reference	11.28 (8.84, 14.41)	Reference	10.96 (8.63, 13.92)	Reference
NH Asian	4.45 (3.54, 5.59)	0.86 (0.72, 1.02)	9.34 (7.28, 11.99)	0.83 (0.66, 1.04)	8.93 (6.47, 12.31)	0.81 (0.60, 1.10)
NH Black	11.05 (8.74, 13.95)	2.13 (1.88, 2.42)	20.78 (16.72, 25.82)	1.84 (1.53, 2.22)	21.23 (16.82, 26.79)	1.94 (1.61, 2.33)
NH Multiple/other	15.86 (11.49, 21.87)	3.06 (2.30, 4.07)	50.47 (21.71, 117.35)	4.47 (2.26, 8.87)	56.26 (22.50, 140.67)	5.13 (2.35, 11.22)
NH NHPI	15.50 (8.42, 28.52)	2.99 (1.65, 5.41)	47.19 (26.59, 83.76)	4.18 (2.28, 7.66)	43.27 (23.66, 79.13)	3.95 (2.11, 7.39)
NH AIAN	11.33 (6.02, 21.35)	2.19 (1.19, 4.02)	27.27 (15.86, 46.90)	2.42 (1.34, 4.37)	29.65 (16.10, 54.63)	2.70 (1.40, 5.22)

NH: Non-Hispanic/Latino; NHPI: Native Hawaiian/Other Pacific Islander; AIAN: American Indian/Alaska Native.

**Table 3:** Incidence per 1,000 and Incidence Rate Ratio (IRR) Estimates for Evaluation 1 (Low Percent of Missingness) that Combined Minnesota and Utah as Target Population

Race/ethnicity (incidence estimates)	^1^Target N=114,793	^2^Complete case analysis					^3^Multiple imputation model 1 (6 individual race variables)					^3^Multiple imputation model 2 (1 multinomial race variable)				
	Incidence (95% CI)	Incidence	Bias	% Bias	Width of CI	Coverage	Incidence	Bias	% Bias	Width of CI	Coverage	Incidence	Bias	% Bias	Width of CI	Coverage
Hispanic/Latino	34.72 (27.07, 44.54)	26.92	−7.81	−22.49	11.10	0.00	34.45	−0.28	−0.81	17.20	1.00	34.06	−0.66	−1.90	16.72	1.00
NH White	7.17 (5.76, 8.93)	5.09	−2.08	−29.01	2.19	0.00	7.17	0.00	0.00	3.24	1.00	7.24	0.07	0.98	3.29	1.00
NH Asian	12.33 (8.86, 17.15)	7.60	−4.73	−38.36	4.80	0.00	12.76	0.44	3.57	11.91	1.00	13.26	0.93	7.54	13.51	1.00
NH Black	30.24 (23.39, 39.09)	18.39	−11.85	−39.19	9.77	0.00	29.91	−0.32	−1.06	17.16	1.00	30.56	0.32	1.06	18.50	1.00
NH Multiple/other	12.57 (10.08, 15.69)	8.40	−4.17	−33.17	3.72	0.00	13.90	1.32	10.50	7.07	1.00	10.06	−2.51	−19.97	4.29	0.18
NH NHPI	64.90 (57.13, 73.73)	43.35	−21.55	−33.20	9.89	0.00	64.30	−0.60	−0.92	12.15	1.00	66.90	2.00	3.08	19.55	1.00
NH AIAN	13.13 (7.95, 21.70)	6.21	−6.92	−52.70	4.39	0.00	12.61	−0.52	−3.96	8.16	1.00	12.28	−0.85	−6.47	8.78	1.00
Race/ethnicity^4^ (IRR estimates)	^1^Target N=114,793	^2^Complete case analysis					^3^Multiple imputation model 1 (6 individual race variables)					^3^Multiple imputation model 2 (1 multinomial race variable)				
	IRR 95% CI	IRR	Bias	% Bias	Width of CI	Coverage	IRR	Bias	% Bias	Width of CI	Coverage	IRR	Bias	% Bias	Width of CI	Coverage
Hispanic/Latino	4.84 (4.16, 5.64)	5.29	0.44	8.09	1.43	1.00	4.81	−0.04	−0.83	1.40	1.00	4.70	−0.14	−2.89	1.31	1.00
NH Asian	1.72 (1.24, 2.38)	1.49	−0.23	−13.37	0.86	1.00	1.78	0.06	3.49	1.73	1.00	1.83	0.11	6.40	1.95	1.00
NH Black	4.22 (3.50, 5.08)	3.61	−0.61	−14.45	1.48	1.00	4.17	−0.04	−0.95	1.85	1.00	4.22	0.00	0.00	2.02	1.00
NH Multiple/other	1.75 (1.54, 2.00)	1.65	−0.10	−5.71	0.46	1.00	1.94	0.18	10.29	0.77	1.00	1.39	−0.37	−21.14	0.48	0.00
NH NHPI	9.05 (7.55. 10.85)	8.51	−0.54	−5.97	3.10	1.00	8.97	−0.08	−0.88	3.19	1.00	9.23	0.18	1.99	3.40	1.00
NH AIAN	1.83 (1.11, 3.01)	1.22	−0.61	−33.33	0.78	0.00	1.76	−0.07	−3.83	1.15	1.00	1.69	−0.14	−7.65	1.23	1.00

Note:

1 The target dataset has no missing values,

2 Complete case analysis is the analysis using only the known information once missing values are induced in the dataset using the missing data model.

3 The multiple imputation model results are based on 10 imputation per replicate.

4 NH White is the reference group.

A total of 100 replicates were performed.

**Table 4:** Incidence per 1,000 and Incidence Rate Ratio (IRR) Estimates for Evaluation 2 (High Percent of Missingness) that Combined Minnesota and Utah as the Target Population

Race/ethnicity (Incidence estimates)	^1^Target N=114,793	^2^Complete case analysis					^3^Multiple imputation model 1 (6 individual race variables)					^3^Multiple imputation model 2 (1 multinomial race variable)				
	Incidence (95% CI)	Incidence	Bias	% Bias	Width of CI	Coverage	Incidence	Bias	% Bias	Width of CI	Coverage	Incidence	Bias	% Bias	Width of CI	Coverage
Hispanic/Latino	34.72 (27.07, 44.54)	19.63	−15.10	−43.49	26.70	1.00	35.21	0.49	1.41	17.63	1.00	34.58	−0.15	−0.43	17.91	1.00
NH White	7.17 (5.76, 8.93)	2.25	−4.92	−68.62	2.75	0.00	6.90	−0.27	−3.77	3.20	1.00	7.08	−0.09	−1.26	3.28	1.00
NH Asian	12.33 (8.86, 17.15)	2.23	−10.10	−81.91	5.00	0.00	16.13	3.80	30.82	29.18	1.00	18.16	5.83	47.28	35.58	1.00
NH Black	30.24 (23.39, 39.09)	4.38	−25.86	−85.52	4.85	0.00	27.06	−3.18	−10.52	22.49	1.00	28.83	−1.41	−4.66	26.13	1.00
NH Multiple/other	12.57 (10.08, 15.69)	3.28	−9.29	−73.91	4.29	0.00	22.15	9.58	76.21	11.17	0.00	9.29	−3.28	−26.09	4.58	0.01
NH NHPI	64.90 (57.13, 73.73)	37.38	−27.53	−42.42	18.58	0.00	61.49	−3.41	−5.25	9.46	0.95	67.87	2.97	4.58	19.78	1.00
NH AIAN	13.13 (7.95, 21.70)	8.28	−4.85	−36.94	15.49	1.00	10.23	−2.90	−22.09	14.24	1.00	10.31	−2.82	−21.48	14.22	1.00
Race/ethnicity^4^ (IRR estimates)	^1^Target N=114,793	^2^Complete case analysis					^3^Multiple imputation model 1 (6 individual race variables)					^3^Multiple imputation model 2 (1 multinomial race variable)				
	IRR (95% CI)	IRR	Bias	% Bias	Width of CI	Coverage	IRR	Bias	*%* Bias	Width of CI	Coverage	IRR	Bias	% Bias	Width of CI	Coverage
Hispanic/Latino	4.84 (4.16, 5.64)	8.74	3.89	80.37	4.57	0.00	5.11	0.26	5.37	1.57	1.00	4.88	0.04	0.83	1.54	1.00
NH Asian	1.72 (1.24, 2.38)	0.99	−0.73	−42.44	1.20	0.83	2.34	0.62	36.05	4.60	1.00	2.57	0.85	49.42	5.48	1.00
NH Black	4.22 (3.50, 5.08)	1.95	−2.27	−53.79	1.94	0.00	3.92	−0.29	−6.87	2.81	1.00	4.07	−0.15	−3.55	3.21	1.00
NH Multiple/other	1.75 (1.54, 2.00)	1.46	−0.29	−16.57	0.43	0.10	3.21	1.46	83.43	1.17	0.00	1.31	−0.44	−25.14	0.42	0.00
NH NHPI	9.05 (7.55, 10.85)	16.63	7.58	83.76	13.12	0.00	8.92	−0.14	−1.55	3.82	1.00	9.59	0.53	5.86	3.80	1.00
NH AIAN	1.83 (1.11, 3.01)	3.69	1.86	101.64	7.85	1.00	1.48	−0.35	−19.13	2.09	1.00	1.46	−0.38	−20.77	2.03	1.00

Note:

1 The target dataset has no missing values,

2 Complete case analysis is the analysis using only the known information once missing values are induced in the dataset using the missing data model.

3 The multiple imputation model results are based on 10 imputations per replicate.

4 NH White is the reference group.

A total of 100 replicates were performed.
