# Supplementary material for: Multiple Imputation of Missing Race and Ethnicity in CDC COVID-19 Case-Level Surveillance Data
Source: Int J Stat Med Res. Author manuscript; Available in PMC 2023 Jan 28. (PMC8967240; doi:10.6000/1929-6029.2022.11.01)
Supplement: supplemental material [file NIHMS1781441-supplement-supplemental_material.pdf]

SUPPLEMENTAL MATERIAL

Part 1. Evaluation Study Procedures

This section contains detailed information on the setup of the evaluation study, using Evaluation 1 (low percent of missingness generated from IA propensity models) as an example. Evaluation 2 follows the same procedure, except using the PA data to develop the propensity models. The procedures are described below.

(1) Step 1: Develop the propensity model for missing race using a logistic regression model with IA data:

$$\text{logit (P (race is missing))} = \text{beta0} + \text{X1*beta1}$$

Covariates considered include age, sex and 18 county level variables (Table **1**) while the final model contains only significant predictors ($P<=0.05$). Let X1 denote a vector of variables included in the final model, beta0 is the intercept, and beta1 is a vector of parameter estimates.

(2) Step 2: Develop the propensity model for missing ethnicity using a logistic regression model with IA data:

$$\text{logit (P (ethnicity is missing))} = \text{gamma0} + \text{X2*gamma1}$$

Covariates considered include age, sex and 18 county level variables while the final model contains only significant predictors ($P<=0.05$). Let X2 denote a vector of variables included in the final model, gamma0 is the intercept and gamma2 is a vector of parameter estimates.

(3) Step 3: Calculate propensity of missing race for the target population. Apply the propensity model on race from step (1) to the MN/UT data, the propensity of missing race (P1) for each person in the MN/UT data is:

$$\text{P1} = \text{exp (beta0 + X1*beta1) / (1+exp (beta0 + X1*beta1))}$$

Where X1 are the observed values from the MN/UT data, beta0 and beta1 are parameter estimates from Step 1.

(4) Step 4: Calculate propensity of missing ethnicity for the target population. Apply the propensity model on ethnicity from step (2) to the MN/UT data, the propensity of missing ethnicity (P2) for each person is:

$$\text{P2} = \text{exp (gamma0 + X2*gamma1) / (1+exp (gamma0 + X2*gamma1))}$$

Where X2 are the observed values from MN/UT data, and gamma0 and gamma1 are parameter estimates from Step 2.

(5) Step 5: Generate two random numbers U1 and U2. For each person in MN/UT, generate two random numbers from a Uniform (0, 1) distribution.

(6) Step 6: Generate missing data on race in MN/UT data. Compare P1 to U1 to decide if a person has a missing value on race, e.g., if $P1 < U1$ then race is missing.

(7) Step 7: Generate missing data on ethnicity in MN/UT data. Compare P2 to U2 to decide if a person has a missing value on ethnicity, e.g., if $P2 < U2$ then ethnicity is missing.

(8) Step 8: Repeated Steps 5-7 100 times to generate 100 replicates.

(9) Step 9: Apply the imputation model to the 100 replicates generated from Step 8 with missing race and ethnicity. For each replicate, 10 imputations were conducted.

Part 2. Sensitivity analysis on missing data mechanism - not missing at random (NMAR) missingness

The evaluation study described in Section 3 assumes the missing data mechanism is missing at random, where the missingness of race and ethnicity depends on the observed covariates. However, it is possible that not all variables related to the missingness are included in the propensity models due to limited individual level information available in the CRF data. Moreover, it is possible that the missingness of race and ethnicity still depends on race and ethnicity after controlling all possible covariates, i.e., not missing at random (NMAR) missingness. In this section, we repeat the evaluation study described in Section 3, with two indicator variables included in the propensity models to generate not missing at random missing data. Specifically, in Evaluation 3 (NMAR, LOW missingness), we include a Hispanic/Latino indicator variable (denoted as HISP-IND, with HISP-IND =1 if a person answered “Yes” to the Hispanic ethnicity question, and HISP-IND =0 if a person answered “No” to the Hispanic ethnicity question) in the IA state ethnicity propensity model, with a coefficient of 0.5; and we include a race indicator variable (denoted as White-IND, with White-IND =1 if a person chose “White” as race, and White-IND =0 if a person didn’t choose White as race) to IA race propensity model with a coefficient of 0.5. In Evaluation 4 (NMAR, High missingness), we included these two variables to the PA state ethnicity propensity model and race propensity model, respectively. With these propensity models, in Evaluation 3, on average, 19.1% subjects have missing data on race, 23.7% subjects have missing data on ethnicity, 34.3% subjects have missing values on combined race/ethnicity; in Evaluation 4, on average, 51.1% subjects have missing data on race, 55.3% subjects have missing data on ethnicity, 68.3% subjects have missing values on combined race/ethnicity.

Evaluation 3 results are shown in Table **S3**. After including HISP-IND and White-IND in the IA propensity models, more Hispanic/Latino and non-Hispanic White subjects have missing values on race/ethnicity information. Based on the complete case analysis, the incidence for Hispanic/Latino is 23.77 per 1,000, compared to 26.92 per 1,000 in Evaluation 1; the incidence for NH White is 4.74 per 1,000 compared to 5.09 per 1,000 in Evaluation 1. Both imputation models reduce biases and improve coverage for all race/ethnic groups, except for NH Multiple/other group, where imputation Model 1 over imputed NH Multiple/other group and imputation Model 2, though yields smaller biases, yield a coverage of 0.57 which is below the 95% nominal level. Excluding NH Multiple/other group, biases using imputation Model 1 range from -2.49 (Hispanic/Latino) to 2.14 (NH NHPI) per 1,000, biases using imputation Model 2 range from -3.03 (Hispanic/Latino) to 6.85 (NH NHPI) per 1,000, where the complete case analysis yields biases ranging from -21.55 (NH NHPI) to -2.43 (NH White) per 1,000; coverage rates are one for both imputation models compared to the coverage rate of zero from the complete case analysis. Accordingly, both imputation models yield smaller biases and better coverage rates in terms of IRR estimates, except the NH Multiple/other group.

Evaluation 4 includes HISP-IND and White-IND in the PA propensity models, the incidence for Hispanic/Latino is 17.32 per 1,000, compared to 19.63 per 1,000 in Evaluation 2; the incidence for NH White is 1.90 per 1,000 compared to 2.25 per 1,000 in Evaluation 2. Complete case analysis yields incidence estimates with biases range from -27.53 (NH NHPI) to -4.85 (NH AIAN) per 1,000, relative biases range from -85.58% (NH Black) to -36.94% (NH AIAN) (Table **S4**). Imputation Model 1 yields incidence estimates with smaller biases compared to the complete cases analysis for all groups except the NH Multiple/other group. Excluding the NH Multiple/other group, biases based on imputation Model 1 range from -3.57 (Hispanic/Latino) to 5.71 (NH-Asian) per 1,000, relative biases range from -20.87% (NH AIAN) to 46.31% (NH-Asian), and coverage rates are above 90%. Imputation Model 2 yields incidence estimates with smaller biases compared to the complete cases analysis for all groups, however, it yields relatively larger bias for NH NHPI group, and low coverage for both NH Multiple/other and NH NHPI groups. Similar patterns are shown in terms of IRR estimates.

In general, imputation Model 1 seems to reduce biases for all race/ethnicity groups except the NH Multiple/other group, in which imputation Model 1 tends to over impute this category. For the remaining race/ethnic groups, imputation Model 1 consistently reduces biases of complete case analysis and improves coverage. Imputation Model 2, though doesn’t over impute NH Multiple/other group, seems to yield estimates with poor coverage for this group, and the biases are slightly larger for the remaining race/ethnicity groups compared to those of imputation Model 1.

Table S1: Population Size, Number of COVID-19 Cases, and Number of Case-Level Data with Missing Race and Ethnicity by State (by 09/25/2020)

State	2018 Population size	Number of known cases in state[1]	Number of cases reported via case report form (CRF)	% completeness	Number of CRF data with missing race	% race missing	Number of CRF data with missing ethnicity	% ethnicity missing
AK	737438	6549	5644	86.18	2501	44.31	3256	57.69
AL	4887871	141757	110685	78.08	34741	31.39	48826	44.11
AR	3013825	73211	67209	91.8	5553	8.26	53292	79.29
AZ	7171646	211660	204905	96.81	74543	36.38	82035	40.04
CA	39557045	766201	754996	98.54	313236	41.49	623223	82.55
CO	5695564	63145	55159	87.35	21204	38.44	14556	26.39
CT	3572665	55386	49669	89.68	24041	48.4	20318	40.91
DC	702455	14790	14595	98.68	72	0.49	2162	14.81
DE	967171	19378	15996	82.55	15876	99.25	7699	48.13
FL	21299325	666507	287684	43.16	69742	24.24	76440	26.57
GA	10519475	300903	157048	52.19	94676	60.28	98669	62.83
HI	1420491	10509	4438	42.23	931	20.98	1230	27.72
IA	3156145	77315	63243	81.8	9249	14.62	8802	13.92
ID	1754208	36489	30944	84.8	10370	33.51	14397	46.53
IL	12741080	270302	261267	96.66	76257	29.19	93239	35.69
IN	6691878	108646	70	0.06	19	27.14	23	32.86
KS	2911510	50870	48426	95.2	8411	17.37	10918	22.55
KY	4468402	59370	11893	20.03	1987	16.71	2144	18.03
LA	4659978	160343	19291	12.03	7661	39.71	13005	67.41
MA	6902149	134035	132740	99.03	28687	21.61	38420	28.94
MD	6042718	119062	85946	72.19	53974	62.8	55798	64.92
ME	1338404	5005	4053	80.98	384	9.47	663	16.36
MI	9995915	126722	115296	90.98	17891	15.52	29471	25.56
MN	5611179	86722	82610	95.26	9843	11.92	13783	16.68
MO	6126452	108334	18128	16.73	938	5.17	4983	27.49
MS	2986530	91935	40790	44.37	2869	7.03	7681	18.83
MT	1062305	9872	8316	84.24	667	8.02	969	11.65
NC	10383620	189576	188806	99.59	41375	21.91	62584	33.15
ND	760077	17230	14764	85.69	13646	92.43	14752	99.92
NE	1929268	39921	17095	42.82	3328	19.47	3152	18.44
NH	1356458	7814	7310	93.55	1042	14.25	805	11.01
NJ	8908520	198361	191943	96.76	78862	41.09	94770	49.37
NM	2095428	27199	21048	77.39	4820	22.9	2531	12.02
NV	3034392	74786	70200	93.87	18851	26.85	24108	34.34
NY	19542209	557976	556673	99.77	346341	62.22	338188	60.75
OH	11689442	141585	132092	93.3	14901	11.28	29365	22.23
OK	3943079	80303	74553	92.84	74544	99.99	74545	99.99
OR	4190713	30060	29150	96.97	3619	12.42	3576	12.27

PA	12807060	147923	143834	97.24	69804	48.53	95940	66.7
RI	1057315	23488	921	3.92	902	97.94	897	97.39
SC	5084127	135446	127147	93.87	37152	29.22	52971	41.66
SD	882235	17686	45	0.25	8	17.78	14	31.11
TN	6770010	178140	166915	93.7	23150	13.87	33975	20.35
TX	28701845	678819	35165	5.18	9464	26.91	7933	22.56
UT	3161105	61631	56355	91.44	5526	9.81	6138	10.89
VA	8517685	138702	126965	91.54	34833	27.44	42543	33.51
VT	626299	1704	1674	98.24	108	6.45	170	10.16
WA	7535591	81198	80285	98.88	31412	39.13	26425	32.91
WI	5813568	100574	64891	64.52	5849	9.01	8170	12.59
WV	1805832	13430	2938	21.88	273	9.29	297	10.11
WY	577737	4652	1300	27.94	204	15.69	360	27.69
Overall	327167439	6723222	4763110	70.85	1706337	35.82	2250211	47.24

[1]Data reported to CDC by state and territorial jurisdictions, including cases reported via case report form (CRF) and aggregated cases without CRF.

Table S2: Means and Frequencies of Variables in the Multiple Imputation Model by Ethnicity

Variables in the imputation model	Hispanic/Latino (N=721,505)		Non-Hispanic/Latino (N=1,791,394)	
	Mean	SE	Mean	SE
Food environment index (raw value)	7.99	0.00	7.73	0.00
Limited access to healthy foods (raw value)	0.05	0.00	0.06	0.00
Log of median household income (raw value)	10.97	0.00	10.96	0.00
Percent Not Hispanic, White alone	53.67	0.02	60.80	0.02
Percent Not Hispanic, Black or African American	13.48	0.01	15.36	0.01
Percent Not Hispanic, American Indian / Alaska Native	0.67	0.00	0.93	0.00
Percent Not Hispanic, Asian alone	5.24	0.01	5.44	0.00
Percent Not Hispanic, Multiple/other race	1.91	0.00	2.04	0.00
Percent Hispanic/Latino	24.87	0.02	15.29	0.01
Percentile ranking for SVI Socioeconomic	0.45	0.00	0.43	0.00
Percentile ranking for SVI Household Composition	0.31	0.00	0.32	0.00
Percentile ranking for SVI Minority Status/Language	0.87	0.00	0.76	0.00
Percentile ranking for SVI Housing / Transportation	0.69	0.00	0.64	0.00
log of population Density	6.64	0.00	6.47	0.00
Adult obesity (raw value)	0.27	0.00	0.28	0.00
Children in poverty (raw value)	0.20	0.00	0.20	0.00
Children in single parent household	0.35	0.00	0.35	0.00
Food insecurity (raw value)	0.13	0.00	0.14	0.00
Age	38.28	0.02	45.78	0.02
Sex (% male)	49.62	0.06	46.49	0.04

Note: P-values <0.001 for all variables for testing if means are equal.

Table S2-A: Means and Frequencies of Variables in the Multiple Imputation Model by Missingness of Race and Ethnicity

Variables in the imputation model	Missingness on race					Missingness on ethnicity				
	Subjects not missing race (N=3,056,773)		Subjects missing race (N=1,706,337)		P-value	Subjects not missing ethnicity (N=2,512,899)		Subjects missing ethnicity (N=2,250,211)		P-value
	Mean	SE	Mean	SE		Mean	SE	Mean	SE	
Food environment index (raw value)	7.81	0.00	8.09	0.00	<0.001	7.81	0.00	8.02	0.00	<0.001
Limited access to healthy foods (raw value)	0.06	0.00	0.05	0.00	<0.001	0.06	0.00	0.05	0.00	<0.001
Log of median household income (raw value)	10.97	0.00	11.04	0.00	<0.001	10.96	0.00	11.03	0.00	<0.001
Percent Not Hispanic, White alone	57.05	0.01	52.00	0.02	<0.001	58.76	0.01	51.32	0.01	<0.001
Percent Not Hispanic, Black or African American	14.29	0.01	14.26	0.01	0.01	14.82	0.01	13.68	0.01	<0.001
Percent Not Hispanic, American Indian / Alaska Native	0.80	0.00	0.94	0.00	<0.001	0.85	0.00	0.84	0.00	0.02
Percent Not Hispanic, Asian alone	5.96	0.00	7.19	0.00	<0.001	5.38	0.00	7.54	0.00	<0.001
Percent Not Hispanic, Multiple/other race	2.05	0.00	2.13	0.00	<0.001	2.01	0.00	2.16	0.00	<0.001
Percent Hispanic/Latino	19.69	0.01	23.35	0.01	<0.001	18.04	0.01	24.30	0.01	<0.001
Percentile ranking for SVI Socioeconomic	0.46	0.00	0.44	0.00	<0.001	0.44	0.00	0.46	0.00	<0.001
Percentile ranking for SVI Household Composition	0.32	0.00	0.27	0.00	<0.001	0.32	0.00	0.29	0.00	<0.001
Percentile ranking for SVI Minority Status/Language	0.80	0.00	0.86	0.00	<0.001	0.79	0.00	0.85	0.00	<0.001
Percentile ranking for SVI Housing / Transportation	0.66	0.00	0.69	0.00	<0.001	0.65	0.00	0.69	0.00	<0.001
log of population Density	6.45	0.00	6.88	0.00	<0.001	6.52	0.00	6.70	0.00	<0.001
Adult obesity (raw value)	0.28	0.00	0.27	0.00	<0.001	0.28	0.00	0.27	0.00	<0.001
Children in poverty (raw value)	0.20	0.00	0.20	0.00	<0.001	0.20	0.00	0.20	0.00	<0.001
Children in single parent household	0.35	0.00	0.34	0.00	<0.001	0.35	0.00	0.34	0.00	<0.001
Food insecurity (raw value)	0.14	0.00	0.12	0.00	<0.001	0.14	0.00	0.13	0.00	<0.001
Age	43.27	0.01	40.78	0.01	<0.001	43.63	0.01	40.99	0.01	<0.001
Sex (% male)	47.35	0.03	49.89	0.04	<0.001	47.39	0.03	49.20	0.03	<0.001

Table S3: Incidence per 1,000 and Incidence Rate Ratio (IRR) Estimates for Evaluation 3 that Combined Minnesota and Utah as Target Population

Race/ethnicity (Incidence estimates)	1Target N=114,793	2Complete case analysis					3Multiple imputation model 1 (6 individual race variables)					3Multiple imputation model 2 (1 multinomial race variable)				
	Incidence (95% CI)	Incidence	Bias	% Bias	Width of CI	Coverage	Incidence	Bias	% Bias	Width of CI	Coverage	Incidence	Bias	% Bias	Width of CI	Coverage
Hispanic/Latino	34.72 (27.07, 44.54)	23.77	-10.95	-31.54	9.41	0.00	32.23	-2.49	-7.17	15.39	1.00	31.70	-3.03	-8.73	14.51	1.00
NH White	7.17 (5.76, 8.93)	4.74	-2.43	-33.89	2.11	0.00	7.14	-0.03	-0.42	3.38	1.00	7.27	0.10	1.39	3.50	1.00
NH Asian	12.33 (8.86, 17.15)	7.60	-4.73	-38.36	4.80	0.00	13.33	1.00	8.11	13.35	1.00	14.19	1.87	15.17	15.29	1.00
NH Black	30.24 (23.39, 39.09)	18.39	-11.85	-39.19	9.77	0.00	31.55	1.31	4.33	19.32	1.00	32.88	2.64	8.73	21.29	1.00
NH Multiple/other	12.57 (10.08, 15.69)	8.40	-4.17	-33.17	3.72	0.00	18.10	5.53	43.99	9.40	0.00	10.21	-2.36	-18.77	4.35	0.57
NH NHPI	64.90 (57.13, 73.73)	43.35	-21.55	-33.20	9.89	0.00	67.04	2.14	3.30	16.30	1.00	71.75	6.85	10.55	24.67	1.00
NH AIAN	13.13 (7.95, 21.70)	6.21	-6.92	-52.70	4.39	0.00	13.15	0.02	0.15	9.61	1.00	13.36	0.23	1.75	9.88	1.00
Race/ethnicity4 (IRR estimates)	1Target N=114,793	2Complete case analysis					3Multiple imputation model 1 (6 individual race variables)					3Multiple imputation model 2 (1 multinomial race variable)				
	IRR (95% CI)	IRR	Bias	% Bias	Width of CI	Coverage	IRR	Bias	% Bias	Width of CI	Coverage	IRR	Bias	% Bias	Width of CI	Coverage
Hispanic/Latino	4.84 (4.16, 5.64)	5.01	0.17	3.51	1.25	1.00	4.51	-0.33	-6.82	1.16	1.00	4.36	-0.48	-9.92	1.05	1.00
NH Asian	1.72 (1.24, 2.38)	1.60	-0.12	-6.98	0.92	1.00	1.87	0.15	8.72	1.96	1.00	1.95	0.23	13.37	2.21	1.00
NH Black	4.22 (3.50, 5.08)	3.88	-0.34	-8.06	1.62	1.00	4.42	0.20	4.74	2.34	1.00	4.52	0.30	7.11	2.58	1.00
NH Multiple/other	1.75 (1.54, 2.00)	1.77	0.02	1.14	0.51	1.00	2.53	0.78	44.57	1.11	0.00	1.40	-0.35	-20.00	0.53	0.02
NH NHPI	9.05 (7.55, 10.85)	9.14	0.09	0.99	3.36	1.00	9.39	0.33	3.65	3.43	1.00	9.87	0.81	8.95	3.75	1.00
NH AIAN	1.83 (1.11, 3.01)	1.31	-0.52	-28.42	0.86	0.31	1.84	0.01	0.55	1.39	1.00	1.84	0.01	0.55	1.41	1.00

Note: [1]The target dataset has no missing values, [2]Complete case analysis is the analysis using only the known information once missing values are induced in the dataset using the missing data model. [3]The multiple imputation model results are based on 10 imputations per replicate. [4]NH White is the reference group. A total of 100 replicates were performed.

Table S4: Incidence per 1,000 and Incidence Rate Ratio (IRR) Estimates for Evaluation 4 that Combined Minnesota and Utah as Target Population

Race/ethnicity (Incidence estimates)	[1]Target N=114,793	[2]Complete case analysis					[3]Multiple imputation model 1 (6 individual race variables)					[3]Multiple imputation model 2 (1 multinomial race variable)				
	Incidence (95% CI)	Incidence	Bias	% Bias	Width of CI	Coverage	Incidence	Bias	% Bias	Width of CI	Coverage	Incidence	Bias	% Bias	Width of CI	Coverage
Hispanic/Latino	34.72 (27.07, 44.54)	17.32	-17.40	-50.12	26.40	0.98	31.16	-3.57	-10.28	19.05	1.00	31.44	-3.28	-9.45	18.44	1.00
NH White	7.17 (5.76, 8.93)	1.90	-5.27	-73.50	2.38	0.00	6.62	-0.55	-7.67	3.19	1.00	6.91	-0.26	-3.63	3.34	1.00
NH Asian	12.33 (8.86, 17.15)	2.23	-10.10	-81.91	5.00	0.00	18.04	5.71	46.31	31.85	1.00	20.60	8.27	67.07	39.20	1.00
NH Black	30.24 (23.39, 39.09)	4.38	-25.86	-85.52	4.85	0.00	31.91	1.67	5.52	26.20	1.00	34.43	4.19	13.86	30.97	1.00
NH Multiple/other	12.57 (10.08, 15.69)	3.28	-9.29	-73.91	4.29	0.00	32.40	19.83	157.76	15.86	0.00	9.08	-3.49	-27.76	4.52	0.01
NH NHPI	64.90 (57.13, 73.73)	37.38	-27.53	-42.42	18.58	0.00	69.14	4.23	6.52	11.36	0.91	78.76	13.86	21.36	24.68	0.07
NH AIAN	13.13 (7.95, 21.70)	8.28	-4.85	-36.94	15.49	1.00	10.39	-2.74	-20.87	14.17	1.00	10.50	-2.64	-20.11	14.14	1.00
Race/ethnicity[4] (IRR estimates)	[1]Target N=114,793	[2]Complete case analysis					[3]Multiple imputation model 1 (6 individual race variables)					[3]Multiple imputation model 2 (1 multinomial race variable)				
	IRR (95% CI)	IRR	Bias	% Bias	Width of CI	Coverage	IRR	Bias	% Bias	Width of CI	Coverage	IRR	Bias	% Bias	Width of CI	Coverage
Hispanic/Latino	4.84 (4.16, 5.64)	9.11	4.27	88.22	5.72	0.00	4.71	-0.14	-2.89	1.74	1.00	4.55	-0.29	-5.99	1.63	1.00
NH Asian	1.72 (1.24, 2.38)	1.17	-0.55	-31.98	1.45	1.00	2.72	1.00	58.14	5.29	1.00	2.98	1.26	73.26	6.23	1.00
NH Black	4.22 (3.50, 5.08)	2.30	-1.91	-45.26	2.40	0.00	4.82	0.60	14.22	3.65	1.00	4.98	0.76	18.01	4.16	1.00
NH Multiple/other	1.75 (1.54, 2.00)	1.73	-0.03	-1.71	0.54	1.00	4.89	3.14	179.43	2.17	0.00	1.31	-0.44	-25.14	0.47	0.00
NH NHPI	9.05 (7.55, 10.85)	19.65	10.60	117.13	16.13	0.00	10.44	1.39	15.36	5.16	1.00	11.40	2.35	25.97	4.92	0.12
NH AIAN	1.83 (1.11, 3.01)	4.36	2.52	137.70	9.33	1.00	1.57	-0.26	-14.21	2.17	1.00	1.52	-0.31	-16.94	2.07	1.00

Note: [1]The target dataset has no missing values, [2]Complete case analysis is the analysis using only the known information once missing values are induced in the dataset using the missing data model. [3]The multiple imputation model results are based on 10 imputations per replicate. [4]NH White is the reference group. A total of 100 replicates were performed.
